# The Roles of Motilin and Ghrelin in Gastrointestinal Motility

**DOI:** 10.1155/2010/820794

**Published:** 2010-02-03

**Authors:** Tetsuro Ohno, Erito Mochiki, Hiroyuki Kuwano

**Affiliations:** Department of General Surgical Science, Gunma University Graduate School of Medicine, 3-39-22 Showa-machi, Maebashi 371-8511, Japan

## Abstract

In structure, ghrelin resembles motilin. The two peptides are considered to be members of the motilin-ghrelin peptide family. Motilin is considered to be an endocrine regulator of the interdigestive migrating contractions, the fasted motor pattern in the gastrointestinal (GI) tract. It has been reported that ghrelin stimulates GI motility. The gastrokinetic capacity of ghrelin has been well documented in the rodent. However, there have been few positive reports of the gastrokinetic capacity of ghrelin in dogs. Some reports with human subjects have shown that an i.v. ghrelin injection accelerated gastric emptying of a meal and improved meal-related symptoms. These results suggest that ghrelin has potential as a prokinetic. However, it seems unlikely that plasma ghrelin would play a physiological role in these digestive physiological events and stimulate gastric emptying, as these outcomes would appear to be in contradiction with the suppression of the endogenous release of ghrelin after eating. The physiological roles of ghrelin need to be clarified.

## 1. Introduction

Ghrelin is a 28-amino-acid peptide predominantly produced by endocrine cells in the oxyntic mucosa of the stomach as an endogenous ligand for the growth hormone (GH) secretagogue receptor [1–4]. Initially, ghrelin was identified as having properties related to the release of GH [5]. Studies have shown that the infusion of ghrelin increases circulating plasma GH in rodents and humans [6–10]. However, other actions of ghrelin have emerged, such as its effects on the glucose metabolism and insulin release [11, 12], cardiovascular actions [13, 14], and food intake and control of energy balance [15, 16].

 Structurally, ghrelin resembles motilin. Motilin is a 22-amino-acid peptide synthesized from endocrine cells of the duodeno-jejunal mucosa. Motilin and ghrelin precursors share almost 50% similarity in their amino-acid sequences, and the receptors of both peptides are part of the same family of G protein-coupled receptors and share 53% overall amino-acid sequence identity [17]. Based upon their structural similarity, the two peptides are now considered to be members of the new motilin-ghrelin peptide family.

Motilin regulates the interdigestive migrating contractions (IMC), the fasted motor pattern in the gastrointestinal (GI) tract [18]. Motilin plasma levels increase cyclically every 90–120 minutes during the interdigestive fasting period, and this cyclical release of motilin disappears after ingestion of a meal. These cyclical peaks of plasma motilin are synchronized to strong peristaltic contractions initiated from the stomach and migrating to the duodenum and small intestine. This pattern of migrating waves is known as the phase III contraction of IMC.

Ghrelin has also been reported to stimulate GI motility [19–22]. Ghrelin administration induces phase III-like contractions in the rat stomach [20]. Ghrelin also induces premature phase III contractions of IMC in the human stomach [21]. Vantrappen et al. [23] reported that motilin induces phase III contractions at a lower dose than ghrelin. On the other hand, high doses of motilin [24] and low doses of ghrelin [5] stimulate GH secretion. As indicated by Peeters [25], these results suggest that both peptides may cross-react with their receptors.

Endogenous ghrelin has been reported to be involved in mediating phase III-like contractions in the stomach of rats [26] and mice [27]. However, we revealed that ghrelin administration did not stimulate GI motility in conscious dogs [28]. Whether ghrelin activates GI motility in dogs and humans is controversial. This review focuses on the capacity of ghrelin to act on GI motility and compares the findings with those of motilin mainly in the dog.

## 2. Motilin and Gastrointestinal Motility

**Figure 1:**
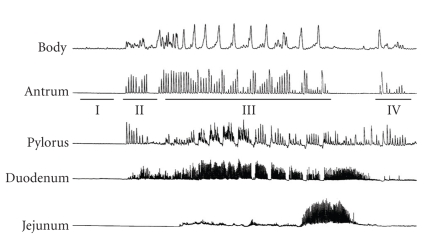
The typical four phases of IMC in the dog. Phase I: quiescence. Phase II: phase of irregular contractile activity. Phase III: intensive rhythmic contractions. Phase IV: rapid decline of activity before complete quiescence.

Because the motilin receptor exists as a pseudogene only in rodents [29, 30], studies on motilin regarding GI motility in animal models are scarce and have been limited to dogs. Motilin is considered to be a unique hormone playing a role in the interdigestive period, rather than, as in the case of most hormones, in the postprandial period. In the interdigestive state, GI motility is characterized by cycling IMC originating in the stomach and propagating along the small intestine. IMCs are assumed to have an important housekeeping role by forcefully pushing the content of the gut forward while cleaning the bowel of debris and bacteria that would otherwise accumulate and lead to bacterial overgrowth and compromise nutrient absorption from the small intestine, resulting in the sensation of hunger. The four phases of IMC were first described by Szurszewski [31]. Figure 1 shows the typical four phases of IMC in the dog. Phase I is quiescence. Phase II is irregular contractile activity. Phase III is characterized by intense, rhythmic contractions starting in the lower esophageal sphincter (LES) and stomach and migrating down the small bowel to the terminal ileum. In Phase IV, the activity rapidly declines until complete quiescence. Soon after motilin's discovery, it was suggested that motilin induces hunger contractions [32]. Itoh et al. [33] showed that the exogenous administration of motilin initiates premature phase III contractions in the stomach that are quite similar to the spontaneously occurring phase III contractions in dogs. This notion is supported by a study by Peeters et al. [34], who described phase III motor activities starting in the stomach or the upper duodenum that are associated with plasma motilin peaks, and another by Lee et al. [35], who reported that immunoneutralization of circulating motilin suppresses phase III contractions.

Many observations suggest the presence of motilin receptors on smooth muscle cells and on neurons of the GI tract. Itoh et al. [36] found that the effect of motilin on phase III activity in dogs was blocked by a 5-hydroxytryptamine-3 (5-HT_3_) antagonist. This finding suggests that the motilin-induced signal may be mediated via 5-HT_3_ receptors on the vagal afferents. The signal is then transmitted to the stomach via vagal efferents that induce the release of endogenous acetylcholine, since anticholinergic agents block the effect of motilin. On the other hand, in many in vitro studies, the induction of contractility by motilin was observed to be resistant to tetrodotoxin, which suggests that motilin receptors are present on smooth-muscle cells [37–39] and the contractile effects of motilin are mediated through a direct action on smooth muscle cells. Most evidence now points to the existence of motilin receptors on nerves as well as on muscles.

## 3. Ghrelin and Gastrointestinal Motility

As for the case of motilin, Tack et al. [21] demonstrated that, in humans, the administration of ghrelin induces a premature gastric phase III, which is not mediated through the release of motilin. Unlike motilin, ghrelin also induced phase III-like contractions in rats and mice [20, 26, 27]. These observations suggest that while the pharmacological effects of ghrelin were demonstrated, the involvement of ghrelin in the control of normal interdigestive motility was not.

**Figure 2:**
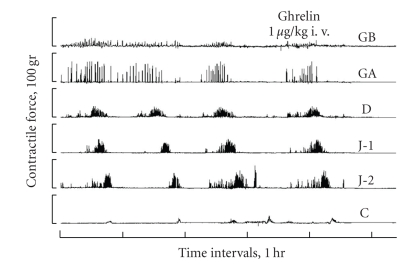
Examples of the effect of canine ghrelin at 1 and 10 *μ*g kg BW^−1^ on the myoelectrical activity in a conscious dog, measured at a gastric body (GB), gastric antrum (GA), duodenum (D), jejunum (J-1 and J-2), and colon (C). Ghrelin did not alter the interval and amplitude of phase III (1 *μ*g kg BW^−1^) (from [28]).

In dogs, we revealed that an i.v. injection of synthesized canine ghrelin did not stimulate motor activity in the digestive tract (Figure 2), although it did stimulate the release of a GH [28]. Kudoh et al. [40] also reported that neither the growth hormone-releasing peptide-2 nor ghrelin evoked GI contractions in the interdigestive state. These results differ from those obtained with studies using rodents. It is reasonable to expect that the action of a peptide will change from species to species. It remains to be determined whether ghrelin could be the surrogate of motilin in rats. The role of endogenous ghrelin in the regulation of phase III-like contractions remains unclear. To date, unlike the case of motilin [34], fluctuation of plasma ghrelin levels in synchrony with phase III activity fronts has not been reported.

## 4. Motilin as a Prokinetic

Motilin has a therapeutic potential as a pharmacological agent in stimulating gastric motility and accelerating gastric emptying of foods. Itoh et al. [41] first showed that erythromycin, a macrolide antibiotic, interacts with the motilin receptor and mimicked the effect of motilin on GI motility during the interdigestive state in dogs. In addition, in humans, erythromycin induces phase III activity [42], and the effect is dose-related in healthy volunteers and patients with diabetic gastroparesis [43]. Erythromycin derivatives devoid of antibiotic activity but with strong affinity for motilin receptors, also called motilides [44], were clinically tested. Clinical studies of ABT-229, one of the motilides, demonstrated the acceleration of gastric emptying in healthy volunteers; however, ABT-229 failed to improve symptoms in patients with functional dyspepsia and diabetic gastroparesis [45, 46]. Disappointing results with ABT-229 decreased the interest in this field of research. However, it was pointed out that several factors associated with the drug (long half-life and/or tachyphylaxis [47] and possible effect on gastric accommodation [48–50]) and the study design (selection of patient population [51]) may have contributed to the negative outcome [52].

A new motilide, GM-611 or mitemcinal, led to promising new results. Takanashi et al. [53] confirmed mitemcinal as a selective and full motilin receptor agonist in in vitro pharmacological studies. Similarly to the case of motilin, intravenous administration of mitemcinal in dogs [54] stimulated interdigestive, as well as digestive, gastroduodenal motor activity, and its effect was blocked by the motilin-receptor antagonist GM-109. Colonic motility in dogs [55] could also be stimulated by mitemcinal given orally. Randomized controlled trials in 392 insulin-requiring diabetics revealed that symptoms attributable to gastroparesis could be ameliorated with 10 mg mitemcinal twice daily than with a placebo and without inducing significant adverse effects [56]. 

## 5. Ghrelin as a Prokinetic

Masuda et al. [19] suggested that ghrelin could stimulate gastric contractions in rats. Trudel et al. [57] documented that ghrelin accelerates gastric emptying and the small intestinal transit of a liquid meal and is a strong prokinetic agent capable of reversing the postoperative gastric ileus in conscious rats. Poitras et al. [58] confirmed that the ghrelin analog RC-1139 is a potent gastrokinetic in rat: it reversed gastric postoperative ileus, even in the presence of opiates.

**Figure 3:**
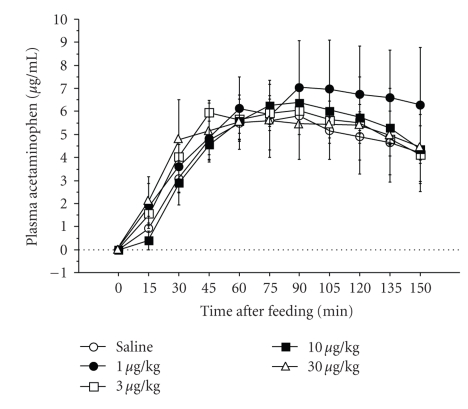
Effects of canine ghrelin on gastric emptying in conscious dogs. Saline and canine ghrelin were administered intravenously. Each symbol represents the mean ± S.E.M. every 15 minutes in three dogs. Differences were not significant (*P* > .05) (from [28]).

The gastrokinetic capacity of ghrelin had been well documented in the rodent. Trudel et al. [59] demonstrated that, as found earlier in rodents, ghrelin accelerates the normal gastric emptying of a meal and was a potent prokinetic agent that improved postoperative gastric ileus in dogs. On the contrary, we [28] showed that an i.v. injection of ghrelin did not accelerate gastric emptying in dogs (Figure 3). To date, encouraging results of the gastrokinetic capacity of ghrelin in dogs are few.

In humans, Binn et al. [60] showed that an i.v. ghrelin injection accelerated gastric emptying of a meal even in the presence of deficient gastric innervation. Tack et al. [61] obtained similar results in which, in idiopathic gastroparesis, the administration of ghrelin enhanced gastric emptying and improved meal-related symptoms. These observations suggest the potential for ghrelin as a prokinetic. TZP-101, a synthetic ghrelin-receptor agonist, has been shown to be an active gastrokinetic agent in rats [62] and has already been tested in humans [63]. However, it seems difficult to believe that plasma ghrelin could play a physiological role in these digestive physiological events. Most evidence indicates that ghrelin plasma levels are high during the fasting period and decrease after meal ingestion. Most GI peptides increase after a meal. Motilin and ghrelin are the only hormones known to decrease in the postprandial period [64]. The observed biological action of the peptide, stimulation of meal gastric emptying, appears to be in contradiction with its endogenous release being suppressed after eating.

## 6. Conclusion

Ghrelin is of great interest, as is motilin, to the GI physiologist. The value of ghrelin as a prokinetic agent may soon be revealed. However, the physiological roles of ghrelin, especially in dogs and humans, need to be clarified.
